# Design of a Micromachined Z-axis Tunneling Magnetoresistive Accelerometer with Electrostatic Force Feedback

**DOI:** 10.3390/mi10020158

**Published:** 2019-02-25

**Authors:** Bo Yang, Binlong Wang, Hongyu Yan, Xiaoyong Gao

**Affiliations:** 1 School of Instrument Science and Engineering, Southeast University, Nanjing 210096, China; 220162760@seu.edu.cn (B.W.); 230189689@seu.edu.cn (H.Y.); 220173252@seu.edu.cn (X.G.); 2 Key Laboratory of Micro-Inertial Instrument and Advanced Navigation Technology, Ministry of Education, Nanjing 210096, China

**Keywords:** accelerometer, tunnel magnetoresistive effect, electrostatic force feedback

## Abstract

This paper presents the design, simulation, fabrication and experiments of a micromachined z-axis tunneling magnetoresistive accelerometer with electrostatic force feedback. The tunneling magnetoresistive accelerometer consists of two upper differential tunneling magnetoresistive sensors, a middle plane main structure with permanent magnetic films and lower electrostatic feedback electrodes. A pair of lever-driven differential proof masses in the middle plane main structure is used for sensitiveness to acceleration and closed-loop feedback control. The tunneling magnetoresistive effect with high sensitivity is adopted to measure magnetic field variation caused by input acceleration. The structural mode and mass ratio between inner and outer proof masses are optimized by the Ansys simulation. Simultaneously, the magnetic field characteristic simulation is implemented to analyze the effect of the location of tunneling magnetoresistive sensors, magnetic field intensity, and the dimension of permanent magnetic film on magnetic field sensitivity, which is beneficial for the achievement of maximum sensitivity. The micromachined z-axis tunneling magnetoresistive accelerometer fabricated by the standard deep dry silicon on glass (DDSOG) process has a device dimension of 6400 μm (length) × 6400 μm (width) × 120 μm (height). The experimental results demonstrate the prototype has a maximal sensitivity of 8.85 mV/g along the z-axis sensitive direction under the gap of 1 mm. Simultaneously, Allan variance analysis illustrate that a noise floor of 86.2 μg/Hz^0.5^ is implemented in the z-axis tunneling magnetoresistive accelerometer.

## 1. Introduction

Numerous applications require high-performance accelerometers including individual navigation and positioning, earthquake early warning systems, the attitude adjustment of micro/nano satellites, oil and gas exploration, and the motion control of micro-autonomous systems [1,2,3]. Various techniques, such as capacitive technology [1], optical principle [4,5], resonance [6,7,8], tunneling [9], piezoelectric [10] and piezoresistive [11] effect, are studied to detect acceleration signals. Among them, the tunneling effect is a potential high precision acceleration measurement method. Traditional tunnel current micro electro mechanical systems (MEMS) accelerometers perform high-resolution detection of displacement based on quantum tunneling effects, which enables high resolution acceleration detection. Compared with other accelerometers, tunneling current accelerometers have the advantages of high sensitivity and wide bandwidth. Many research institutes have implemented extensive research on tunneling tip processing technology, thin film deposition technology, feedback control technology and tunneling current testing technology [12]. The traditional tunneling MEMS accelerometer can obtain extremely high resolution, however, its dynamic range is extremely narrow. The main reason is that the nm gap between the tunneling tip and the substrate needs to be controlled in order to achieve the tunneling effect, which results in limited dynamic range. At the same time, the current fabrication tolerance of micromachining process makes the nm gap hard to process, which makes the traditional tunneling current accelerometer difficult to implement. 

Inspired by the successful application in other commercial products, a new tunneling magnetoresistance technology with ultra-high sensitivity, which surpasses the shortcomings of traditional tunneling current effects, has rapidly penetrated the field of micro-inertial devices in recent years [13]. Phan et al. at the Eindhoven University of Technology present a polydimethylsiloxane (PDMS) based biaxial accelerometer by using the magnetoresistive (MR) detection method [14]. The proof mass of the sensor is a mushroom-shaped polymer magnet, whose minute movement under lateral acceleration is precisely sensed by a set of MR sensors. A sensitivity of 0.32 mV/(V.g) and a noise density of 35 μg/Hz^0.5^ at 5 Hz was obtained in the device. Literature from Pacesetter Inc proposed a magnetoresistive-based position sensor for use in an implantable electrical device [15]. The sensor includes a magnetoresistive sensor made from giant magnetoresistive (GMR) materials and a magnet positioned on a flexible cantilevered beam. The relative movement of the magnet, which originates from the movement of the patient, is detected by the magnetoresistive sensor. The realization of the device is not given in the literature. Ali Alaoui at Crocus Technology SA proposed a MLU (Magnetic Logic Unit) based biaxial accelerometer using a magnetic tunneling junction [16]. An ultra-sensitive displacement sensing device is presented by Olivas et al. of the National Aeronautics and Space Administration (NASA) for use in accelerometers, pressure gauges, temperature transducers [17]. The device comprises a sputter deposited, multilayer, magnetoresistive field sensor with a variable electrical resistance based on an imposed magnetic field. The scheme implements a magnetoresistive structure and gap control using micromachining technology, which already has the basic elements of a miniature magnetoresistive accelerometer. Similarly, a cantilever-based precision force detection MEMS device with magnetoresistance technology was used to measure acceleration information at the Sony Precision Engineering Center in Singapore [18]. In addition, David P. Fries et al. in the University of South Florida has presented a gyroscope based on giant magnetoresistance effect [19]. Currently, most of the above accelerometers are based on the traditional magnetoresistance or giant magnetoresistance (GMR) effects, which limit the further improvement of accelerometer accuracy due to the restriction of displacement sensitivity. The literature [20] has proposed a small tunnel magnetoresistive accelerometer based on 3D printing, however the accelerometer with a large volume and weight is fabricated by traditional macro processing technology. Therefore, the structure is not a true micromechanical accelerometer, and the support cantilever beam of proof mass with three-dimensional structure is also difficult to achieve through micromachining processing technology. Simultaneously, the accelerometer can only measure the input acceleration through open loop detection due to the lack of feedback torque mechanism.

This paper presents a micromachined z-axis tunneling magnetoresistive accelerometer with electrostatic force feedback. The tunneling magnetoresistive accelerometer consists of two upper differential tunneling magnetoresistive sensors, a middle plane main structure with permanent magnetic films and lower electrostatic feedback electrodes. The proof mass with permanent magnet film on the plane main structure is driven to move by the input acceleration, then the caused magnetic field change is measured by tunneling magnetoresistive sensors. Finally, the electrostatic feedback electrodes are used to adjust the proof mass to the equilibrium position, thereby closed loop detection is implemented. In Section 2, the structure principle of the micromachined z-axis tunneling magnetoresistive accelerometer is briefly presented. Then the simulation analysis as well as the measurement and control circuit are given in Section 3 and Section 4. We demonstration the experimental results in Section 5. Concluding remarks are given in the last section. 

## 2. Structure Principle

**Figure 1:**
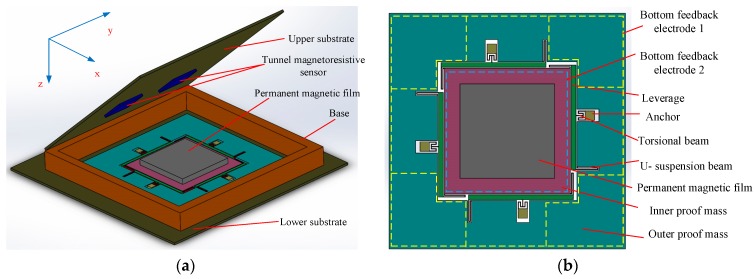
Structural schematic of the micromachined z-axis tunnel magnetoresistive accelerometer: (**a**) The structure layout of tunnel magnetoresistive accelerometer; (**b**) The plane main structure of tunnel magnetoresistive accelerometer.

The cantilever beam structure with proof mass is one of the core components of the micromechanical accelerometer and can directly convert input acceleration into stress or displacement. It has the advantages of a simple structure, high sensitivity and high conversion efficiency. The cantilever beam structure with proof mass has to be innovatively designed in order to be compatible with tunneling magnetoresistive effects for acceleration measure. A structural schematic of the micromachined z-axis tunneling magnetoresistive accelerometer, which consists of upper, middle and lower layers, is shown in Figure 1.

Two tunnel magnetoresistive sensors with opposite sensitive direction (y-axis in the Figure 1a) in the upper substrate which are symmetrically arranged on the sides of the centerline are used to measure the variations of the surrounding magnetic field caused by the input acceleration. The middle layer is the plane main structure of tunnel magnetoresistive accelerometer shown in Figure 1b. The plane main structure is composed of four lever mechanisms, the permanent magnetic film, outer and inner proof mass. The permanent magnetic film is arranged on the inner proof mass. The outer proof mass is connected to the inner proof mass by four leverages. When the acceleration is input along the z-axis, the inertia forces at both ends of the leverage will be unbalanced due to the difference in mass between the outer proof mass and inner proof mass with permanent magnetic film, which cause the inner and outer proof masses to move in opposite directions along the z-axis. Since the gap between the permanent magnetic film and the tunnel magnetoresistive sensor varies due to the input acceleration, there will be a redistribution of the surrounding magnetic field in the tunneling magnetoresistive sensor. Therefore, the input acceleration can be measured indirectly by the output of the tunneling magnetoresistive sensor. The tunneling magnetoresistance effect with high sensitivity is directly generated by the multilayer nano-film layer in the tunneling magnetoresistive sensors, which can be easily achieved by a multi-thin-film deposition process. Therefore, the micromachining requirement for the nano-gap between the tunnel tip and the plane in the traditional tunnel effect is avoided. Simultaneously, the gap between the permanent magnetic film and the tunnel magnetoresistive sensor can be further amplified, which will facilitate the realization of a large dynamic range. Furthermore, two feedback electrodes on the lower substrate, which are arranged under the inner and outer proof masses, are used to achieve an electrostatic force feedback and closed loop control in subsequent operations.

**Figure 2:**
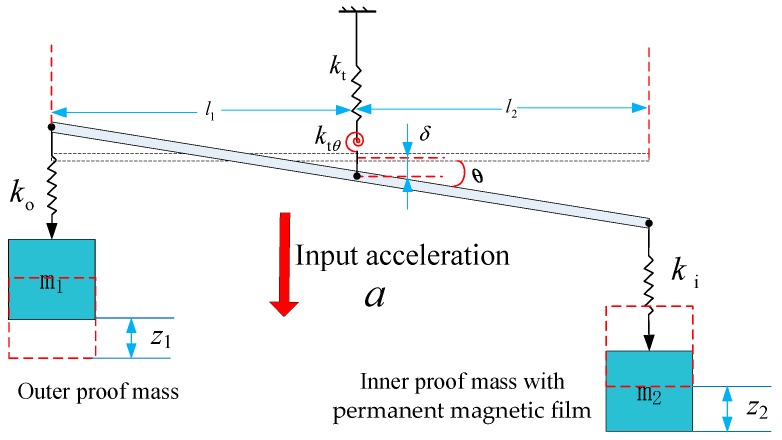
Simplified structural model.

The entire structural model is simplified, as shown in Figure 2. The leverage equations are

(1) $$k_{t}\delta =(m_{1}+m_{2})a$$

(2) $$m_{2}{al}_{2}=m_{1}{al}_{1}+k_{t\theta }\theta$$

(3) $$z_{1}=l_{1}\theta -\delta -\frac{m_{1}a}{k_{o}}$$

(4) $$z_{2}=l_{2}\theta +\delta +\frac{m_{2}a}{k_{i}}$$

where *k*_t_ and *k*_t__θ_ are the stiffness along the z-axis and torsional stiffness of torsional beam respectively, *k*_o_ and *k*_i_ is stiffness of U-suspension beam, *l*_1_ and *l*_2_ are the arm length of leverage, *z*_1_ and *z*_2_ are the displacement of outer and inner proof masses, respectively.

The weighted displacement *z* is

(5) $$z=\frac{m_{1}z_{1}+m_{2}z_{2}}{m_{1}+m_{2}}=(\frac{m_{{}^{{}_{2}}}^{2}l_{2}^{2}-m_{{}^{{}_{1}}}^{2}l_{1}^{2}}{k_{t\theta }(m_{1}+m_{2})}+\frac{m_{2}-m_{1}}{k_{t}}+\frac{m_{{}^{{}_{2}}}^{2}}{k_{i}(m_{1}+m_{2})}-\frac{m_{{}^{1}}^{2}}{k_{o}(m_{1}+m_{2})})a$$

Suppose *k*_i_ = *k*_o_ and *l*_1_ = *l*_2_ = *l,* then

(6) $$z=\frac{G_{m}a}{\omega_{n}^{2}}$$

where *G*_m_ is mass ratio, *ω*_n_ is modal natural frequency of accelerometer along the z-axis, *k_e_* is the equivalent stiffness and

(7) $$G_{m}=\frac{m_{2}-m_{1}}{m_{1}+m_{2}},\;\omega_{n}^{2}=\frac{k_{e}}{m_{1}+m_{2}},\;k_{e}=\frac{1}{\frac{l^{2}}{k_{t\theta }}+\frac{1}{k_{t}}+\frac{1}{k_{i}}}$$

The magnetic field distribution along the y-axis due to a rectangular permanent magnetic film can be expressed approximately as *B*_y_ (*x*, *y*, *z*) [21]. Only the magnetic field distribution along the y-axis is given because the sensitive direction of two tunneling magnetoresistive sensors is the y-axis.

(8) $$B_{y}(x,y,z)=\frac{\mu_{0}M}{4\pi }\ln\frac{F_{2}(-y,x,-z)F_{2}(y,x,z)}{F_{2}(y,x,-z)F_{2}(-y,x,z)}$$

where

(9) $$F_{2}(x,y,z)=\frac{\sqrt{{\left(x+a\right)}^{2}+{\left(y-b\right)}^{2}+{\left(z+c\right)}^{2}}+b-y}{\sqrt{{\left(x+a\right)}^{2}+{\left(y+b\right)}^{2}+{\left(z+c\right)}^{2}}-b-y}$$

*M* is the moment density. *a*, *b* and *c* are half of length along the x-axis, half of width along the y-axis and half of thickness along the z-axis in the rectangular permanent magnetic film, respectively.

The magnetic field distribution along the y direction caused by the displacement movement in the z-axis is simplified as

(10) $$B_{yz}(x,y,z)=k_{Bz}\Delta z+B_{y}(x_{0},y_{0},z_{0})$$

where $k_{Bz}=\frac{\partial B_{y}(x,y,z)}{\partial z}{|}_{\left(x_{0},y_{0},z_{0}\right)}$.

The outputs of the tunnel magnetoresistive sensor are

(11) $$V_{z}\approx k_{v}B_{\mathrm{yz}}(x,y,z)\approx \frac{G_{m}k_{v}k_{Bz}}{\omega_{n}^{2}}a+k_{v}B_{y}(x_{0},y_{0},z_{0})$$

where *k*_v_ is equivalent transform coefficient of tunneling magnetoresistive sensor from magnetic field to voltage. The structure parameters of the tunneling magnetoresistive accelerometer are shown in Table 1.

## 3. Simulation Analysis

**Figure 3:**
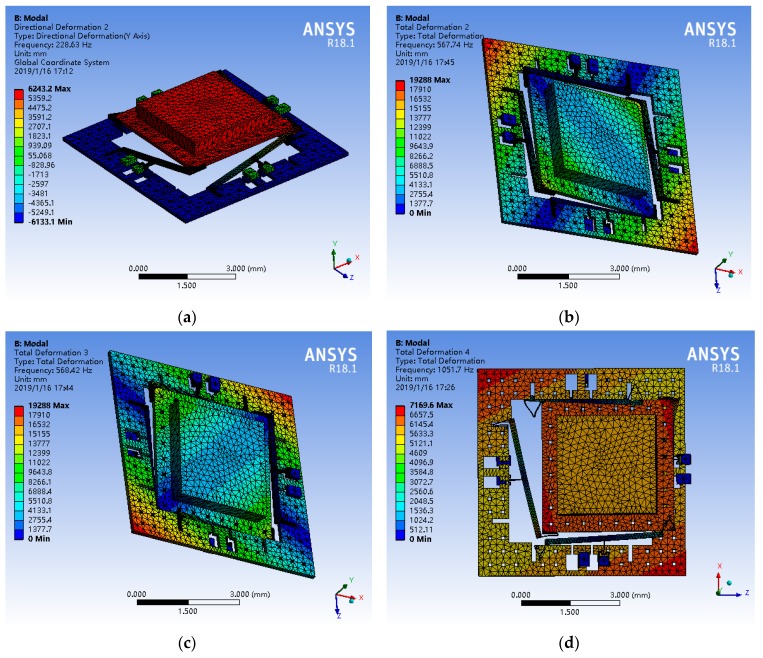
The selected modes of the micromachined z-axis tunneling magnetoresistive accelerometer. (**a**) Linear movement of proof mass along z direction in first mode; (**b**) The off-plane rotation movement of inner proof mass in second mode; (**c**) The off-plane rotation movement of inner proof mass in the third mode; (**d**) The in-plane translational movement of inner proof mass in the fourth mode.

The main structure of the tunneling magnetoresistive accelerometer is simulated by the Ansys in order to verify the structural principle. The modal simulation results are shown in Figure 3. The first mode with a frequency of 228.6 Hz is the sense mode of acceleration along the z-axis. The inner proof mass is pushed to move downward along the z-axis by inertial force, and promote simultaneously the outer proof mass to move upward by the leverage, since the mass of the inner proof mass is greater than that of the outer proof mass. Two interference modes shown in Figure 3b,c are off-plane rotation movement of inner proof mass respectively. The interference mode shown in Figure 3d is the in-plane translational movement of inner proof mass in the fourth mode. Other disturbance modes are shown in Table 2. Simultaneously, the simulation results demonstrate that the mechanical structure of the tunnel magnetoresistive accelerometer has a mechanical sensitivity of 3.05 μm/g in z-axis sense direction.

**Figure 4:**
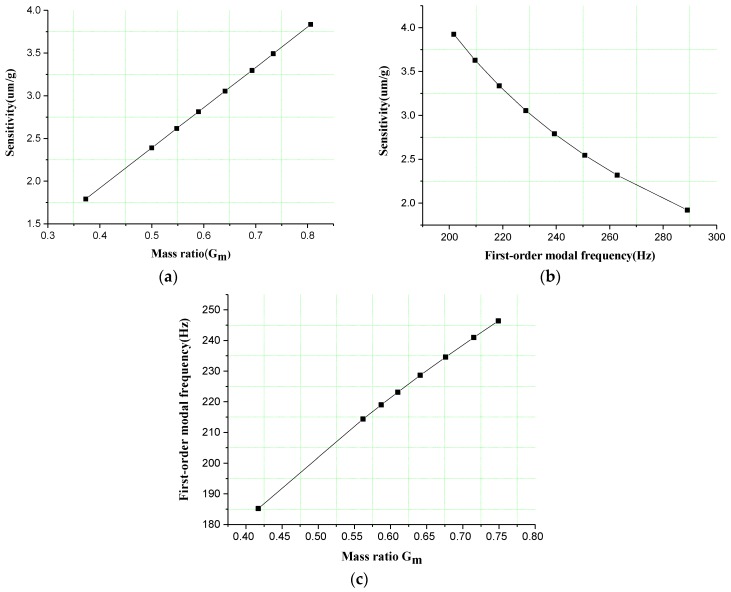
The effect of mass ratio and first-order modal frequency on mechanical sensitivity (**a**) The relationship between mechanical sensitivity and mass ratio in the same first-order modal frequencies; (**b**) The relationship between mechanical sensitivity and first-order modal frequencies in the same mass ratio; (**c**) The relationship between mass ratio and first-order modal frequency in the same mechanical sensitivity.

The effect of mass ratio and first-order modal frequency on mechanical sensitivity is shown in Figure 4. As can be seen from Figure 4a,b, the mechanical sensitivity is clearly proportional to the mass ratio at the same first-order modal frequency and inversely proportional to the first-order modal frequency at the same mass ratio. Simultaneously, the first-order modal frequency is proportional to the mass ratio under the same mechanical sensitivity conditions, shown in Figure 4c. That is, in order to maintain the same mechanical sensitivity, the mass ratio must be increased accordingly when the first-order mode is added. Actually, the increase of the mass ratio is beneficial to enhance the mechanical sensitivity. In the limit case, if m_1_ = 0, the maximum mass ratio can be obtained, which can implement the maximum mechanical sensitivity. However, a compromise design has been made in order to be compatible with the electrostatic torque devices in the structure. Reverse electrostatic forces for closed loop feedback control are implemented by the electrostatic torque devices which are constituted by a pair of differential masses (m_1_ and m_2_) with a pair of differential electrodes and lever mechanisms. In summary, the above simulation results are in good agreement with the above theory, which confirms the correctness of theoretical analysis. 

**Figure 5:**
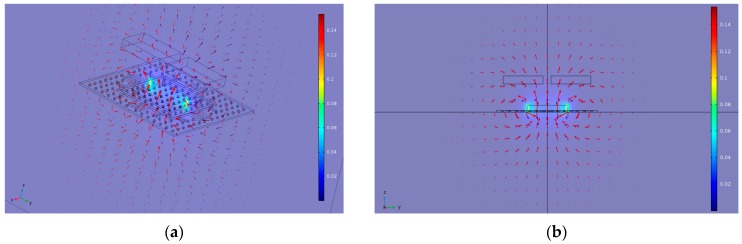
Finite element simulation of magnetic field distribution for permanent magnetic film. (**a**) Three dimensional magnetic field distribution; (**b**) Planar magnetic field distribution in the middle section along the y-direction.

Furthermore, a finite element simulation based on a solid model and a numerical simulation are implemented to analyze the magnetic field characteristics of the entire accelerometer. The physical structure is constructed using Comsol software according to the parameters shown in Table 1. The finite element simulation of magnetic field distribution for permanent magnetic film is shown in Figure 5.

The magnetic field simulation results demonstrate that the magnetic field generated by the miniature rectangular permanent magnetic film is basically similar to the macro magnetic field. And the closer to the permanent magnetic film, the greater the magnetic field strength, as shown in Figure 5a. Simultaneously, the planar magnetic field distribution in the middle section shown in Figure 5b indicates that two tunnel magnetoresistive sensors located directly above the boundary of the permanent magnetic film have opposite magnetic field directions. The sensitive direction of the tunneling magnetoresistive sensor is the y direction. The magnetic field change caused by the displacement in the z direction still has a component in the y direction since the magnetic field direction on the tunnel magnetoresistive sensor is not orthogonal to the y direction, which is the sensitive mechanism of the tunnel magnetoresistive accelerometer.

**Figure 6:**
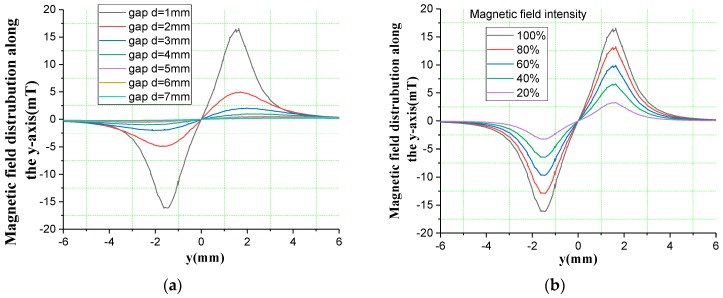

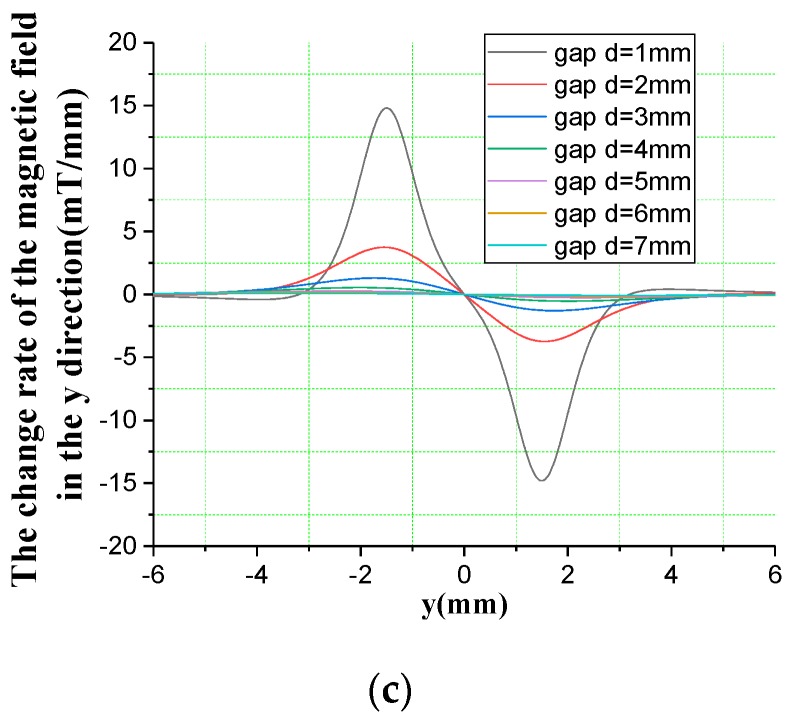
Magnetic field characteristic in different conditions (**a**) Magnetic field distribution along the y-axis in the tunnel magnetoresistive sensor versus different gaps; (**b**) Magnetic field distribution along the y-axis in the tunnel magnetoresistive sensor versus different magnetic field intensity of permanent magnetic film (d = 1 mm); (**c**) The change rate of the magnetic field in the y direction due to a displacement variation in the z direction versus different gaps.

The magnetic field data under different conditions are extracted for further quantitative analysis. The magnetic field distribution in the y direction is mainly considered since the sensitive direction of the tunnel magnetoresistive sensor is the y axis. Figure 6 shows the magnetic field characteristic in different conditions.

Magnetic field intensity along the y-axis in the tunneling magnetoresistive sensor shown in Figure 6a is inversely proportional to the gap between the tunnel magnetoresistive sensor and the permanent magnetic film. The maximum magnetic field intensity is decreased by 77.1 times when the gap is increased from 1 mm to 7 mm. The maximum magnetic field intensity in the tunnel magnetoresistive sensor reached 16.57 mT under a gap of 1 mm. The entire magnetic field intensity cannot approach the saturated area or exceed the measurable range of the tunneling magnetoresistive sensor, otherwise it will cause sensitivity attenuation, saturation and lose the measurement capability in the tunneling magnetoresistive sensor. This will limit the optional gap and shift position of y between the permanent magnetic film and tunneling magnetoresistive sensor. Therefore, the normal magnetic field signal detection can be achieved in the suitable gap and shift position of y by the tunneling magnetoresistive sensor. Figure 6b shows the magnetic field distribution along the y-axis in the tunnel magnetoresistive sensor versus different magnetic field intensities of permanent magnetic film (d = 1 mm). The maximum magnetic field intensity in the tunneling magnetoresistive sensor can significantly reduce by decreasing the magnetic field strength of the magnetic film under the same gap. When the magnetic field strength of the permanent magnetic film is reduced to 20 %, the maximum magnetic field intensity in the tunneling magnetoresistive sensor is decreased from 16.58 mT to 3.31 mT.

Further, a numerical simulation is differentiated to abstract the change rate of the magnetic field due to the displacement variation in the z directions. Apparently, the change rate of the magnetic field in the y direction due to a displacement variation in the z direction decreases as the gap increases, shown in Figure 6c. The maximum change rate of the magnetic field is reduced from 14.8 mT/mm to 0.079 mT/mm when the gap is increased from 1 mm to 7 mm. Simultaneously, the change rate of the magnetic field due to a displacement variation along the z direction in different horizontal shift between the tunnel magnetoresistive sensor and permanent magnetic film is shown in Figure 6c. The change rate of the magnetic field is essentially zero when horizontal shift y = 0, which indicates that the minimal sensitivity in the tunneling magnetoresistive sensor appears at this point. The main reason for this is that the direction of the magnetic field is orthogonal to the sensitive direction of the tunnel magnetoresistive sensor at this point, which is basically consistent with the finite element simulation of Figure 5b. At the same time, when the horizontal shift is equal to 1.5 mm, the change rate of the magnetic field in the y direction has approximately the largest absolute value, which demonstrates that the tunneling magnetoresistive sensor has the greatest sensitivity at the boundary of the permanent magnetic film. This is also the theoretical basis for the layout optimization of the tunneling magnetoresistive sensor.

**Figure 7:**
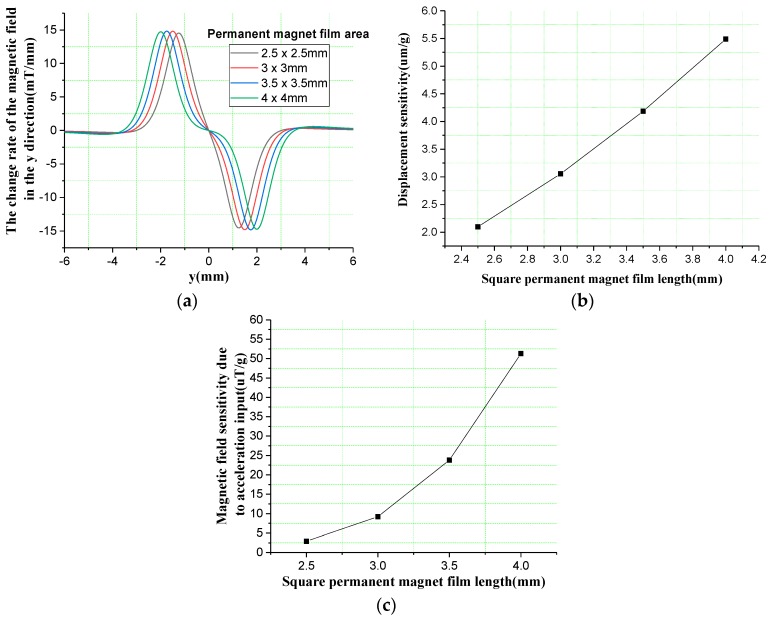
Influence of structure dimension of permanent magnet film on characteristics of the tunneling magnetoresistive accelerometer. (**a**) The change rate of the magnetic field in the y direction due to a displacement variation in the z direction versus different structure dimension of permanent magnet film; (**b**) Mechanical sensitivity versus different structure dimension of permanent magnet film; (**c**) The magnetic field sensitivity in the y direction due to input acceleration in the z direction versus different structure dimension of permanent magnet film (y = 2.5 mm).

The structure dimension of permanent magnetic film not only affects the distribution of the magnetic field, but also affects the mechanical sensitivity. Figure 7 shows the influences of structure dimension of permanent magnetic films on characteristics of the tunneling magnetoresistive accelerometer. Obviously, the variation in the structure dimension of the permanent magnet film causes the maximum change rate of magnetic field to appear at different positions, shown in Figure 7a. However, all the maximum change rates of magnetic field appear almost around the boundary of the permanent magnetic film. At the same time, the mechanical sensitivity of the displacement increases by 2.62 times as the structure dimension of permanent magnetic film widens from 2.5 mm × 2.5 mm to 4 mm × 4 mm, as shown in Figure 7b. Combining the above two effects, the change rate of the magnetic field in the y direction due to input acceleration in the z direction is proportional to the change of structure dimension of the permanent magnetic film, as shown in Figure 7c. As the structure dimension of the permanent magnetic film is enlarged from 2.5 mm × 2.5 mm to 4 mm × 4 mm, the magnetic field sensitivity in the y direction due to input acceleration in the z direction increases by 17.62 times.

## 4. Measurement and Control Circuit

**Figure 8:**
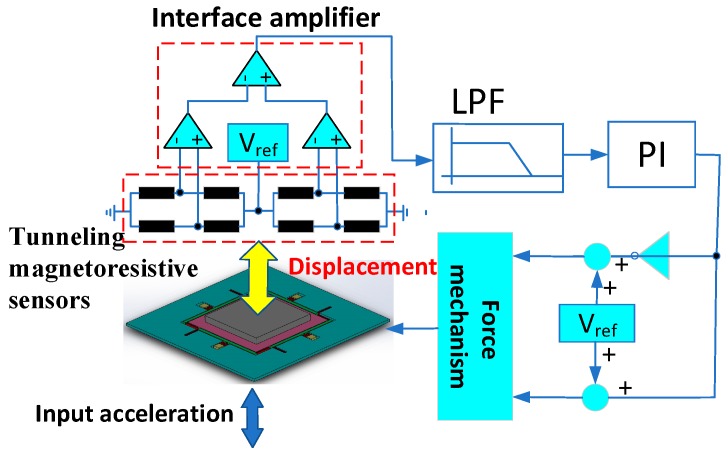
The scheme of measurement and control circuit.

The scheme of the measurement and control circuit is shown in Figure 8. The displacement of the permanent magnetic film due to input acceleration causes the resistance change of the tunnel magnetoresistive sensor. Two resistance bridges are formed by eight tunneling magnetoresistive sensors arranged on the two boundaries of the permanent magnetic film. An interface amplifier circuit consisting of three operational amplifiers is used to measure resistance changes. Then, the signal is filtered by a low pass filter (LPF) and control signals are generated by the proportional-integral (PI) controller. Finally, the output signal of the PI controller is divided into two paths, which are respectively applied to the two feedback electrodes. The feedback correction force is generated by the electrostatic force mechanism to realize the closed-loop detection of the tunneling magnetoresistive accelerometer.

## 5. Experiment

**Figure 9:**
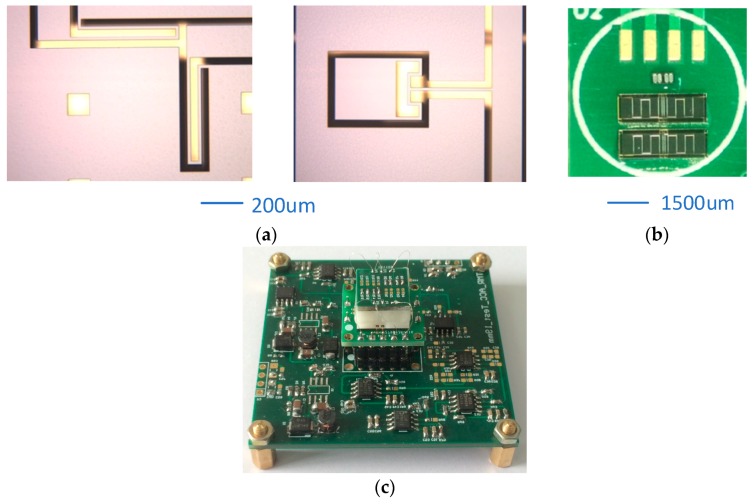
The optical micrograph of micromachined z-axis tunnel magnetoresistive accelerometer. (**a**) The plane main structure of tunnel magnetoresistive accelerometer; (**b**) Commercial tunnel magnetoresistive sensor; (**c**) Micro-assembled overall structure.

The micromachined z-axis tunneling magnetoresistive accelerometer is fabricated by a standard deep dry silicon on glass (DDSOG) process in order to verify the theoretical analysis and evaluate the characteristics. Three masks are used to implement the fabrication of the micromachined z-axis tunnel magnetoresistive accelerometer. The first mask is utilized to pattern and expose the bonding anchors in a monocrystalline wafer with 200 μm thickness by lithography, and steps of bonding anchors with 10 μm height are etched by the deep reactive ion etching (DRIE). The electrode wires and pads with a Cr/Ti/Au stack layer are established by the sputtering process with the second mask in a Pyrex glass substrate with 500 μm thickness. Then the silicon wafer and the Pyrex glass wafer are linked together by the electrostatic anodic bonding process, and the silicon wafer is thinned to 120 μm thickness by a wet etching process with KOH solution. Subsequently, the mechanical structure is lithographically patterned and etched to release by the DRIE with the third mask. Finally, two commercial tunneling magnetoresistive sensors and a permanent magnetic film are patterned and installed by the micro-assembly process. The performance of the prototype is sensitive to the distance between the permanent magnetic film and the tunnel magnetoresistive sensor. We adjust the gap between the permanent magnetic film and the tunnel magnetoresistive sensor through the base. The base is realized by 3D printing technology, and its precision can be controlled within several tens of μm. The tunneling magnetoresistive sensors adopts the commercial linear sensor of TMR9001 in Multi-Dimension Technology [22]. The optical micrograph of micromachined z-axis tunnel magnetoresistive accelerometer is shown in Figure 9. The fabricated plane main structure of tunnel magnetoresistive accelerometer has a dimension of 6400 μm (length) × 6400 μm (width) × 120 μm (height) with a permanent magnet film of 3000 μm (length) × 3000 μm (width) × 500 μm (height).

**Figure 10:**
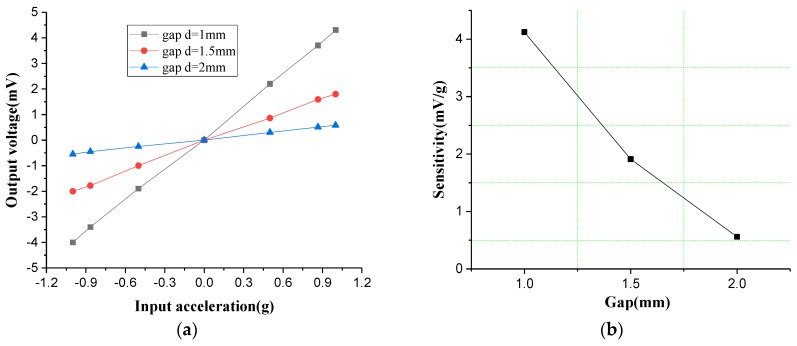
Acceleration input and output response characteristics under different gaps in the tunneling magnetoresistive accelerometer. (**a**) Input acceleration versus output voltage under different gaps; (**b**) The sensitivity versus gap.

In order to fully evaluate the performance of tunnel magnetoresistive accelerometers, system experiments under various conditions are implemented. We test the acceleration input and output response characteristics under different gaps in the tunneling magnetoresistive accelerometer, shown in Figure 10. The experiment results demonstrate that the signal sensitivity is inversely proportional to gap variation. When the gap is reduced from 2 mm to 1 mm, the signal sensitivity is increased by 7.37 times from 0.559 mV/g to 4.12 mV/g. This confirms that the decrease of the gap between the tunneling magnetoresistive sensors and permanent magnetic film can significantly increase the change rate of the magnetic field due to a displacement variation along the z direction, which is consistent with the previous simulation analysis.

**Figure 11:**
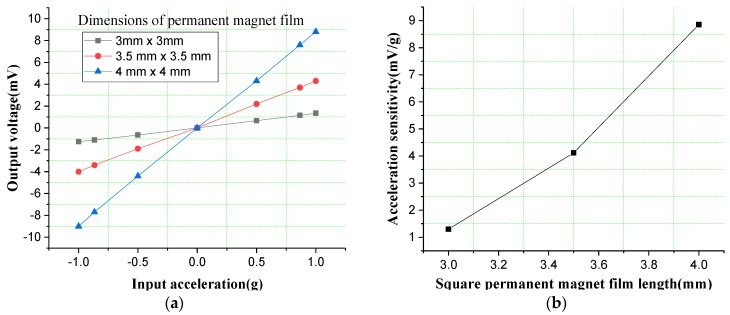
Acceleration input and output response characteristics under different dimensions of permanent magnetic film in the tunneling magnetoresistive accelerometer. (**a**) Input acceleration versus output voltage under different dimensions of permanent magnetic film; (**b**) The sensitivity versus dimensions of permanent magnet film.

Acceleration input and output response characteristics experiments under different dimensions of permanent magnetic film are implemented to analyze the influence on the performance of the tunneling magnetoresistive accelerometer, shown in Figure 11. The experiment results illustrate that the signal sensitivity is proportional to the dimension of permanent magnetic film. When the dimension of the permanent magnetic film is widened from 3 mm × 3 mm to 4 mm × 4 mm, the sensitivity is increased by 6.81 times from 1.30 mV/g to 8.85 mV/g. This demonstrates that the area amplification of the magnetic field can significantly improve the mechanical displacement sensitivity and the magnetic field change rate due to a displacement variation along the z direction, and ultimately improve the signal sensitivity of the tunneling magnetoresistive accelerometer, which is consistent with the aforementioned simulation analysis.

**Figure 12:**
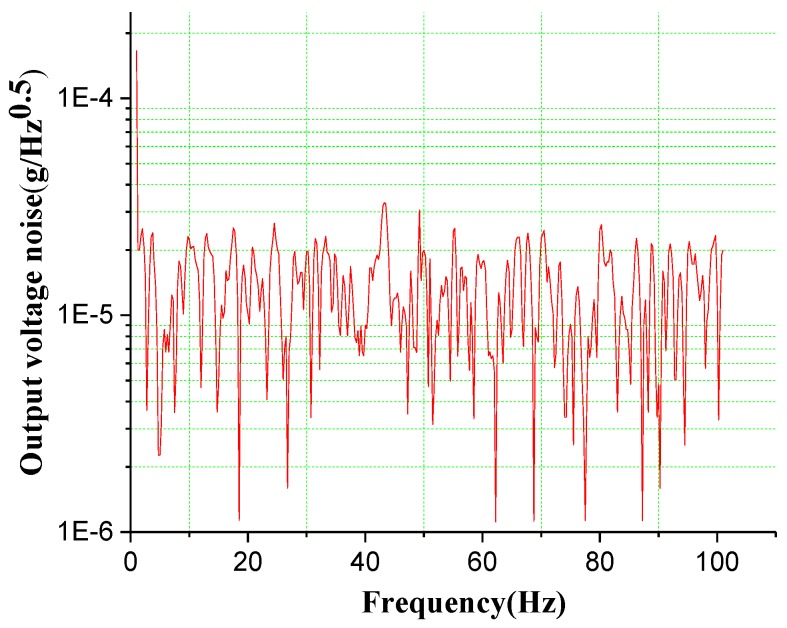
Output voltage noise spectrum of z-axis tunneling magnetoresistive accelerometer.

Finally, the stability and noise performance measures of the z-axis tunneling magnetoresistive accelerometer are implemented to evaluate the performance of the prototype. In order to optimize system performance, the gap and shift of the tunneling magnetoresistive sensor are slightly adjusted for maximum sensitivity along z-axis sensitive direction. The output voltage noise spectrum of prototype is shown in Figure 12. 

**Figure 13:**
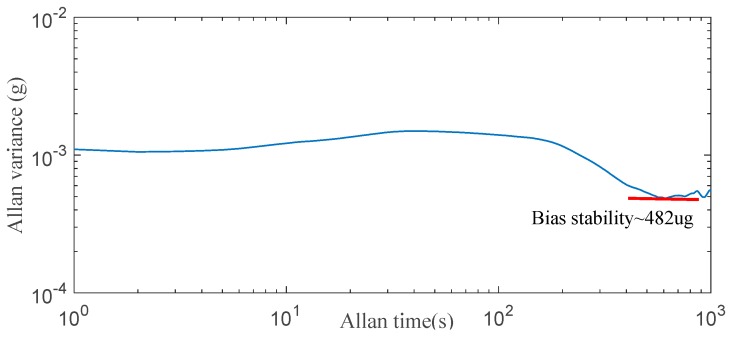
Drift stability curve of z-axis tunneling magnetoresistive accelerometer based on Allan variance.

Experimental results demonstrate that the tunneling magnetoresistive accelerometer has a noise floor of 86.2 μg/Hz^0.5^ along z-axis sensitive direction. The noise comes from mechanical thermal noise of proof mass, resistance thermal noise of tunnel magnetoresistive sensor and circuit noise of the interface amplifier. The noisy force generator of mechanical thermal noise corresponds to an equivalent input acceleration *a*_n_ = (4*k*_B_*Tξ*)^0.5^/(9.8*m*) [23]. Suppose the Boltzman’s constant *k*_B_ = 1.38 × 10^−23^ J/K, the absolute temperature *T* = 300 K, the viscous damping coefficient *ξ* = 0.5 N/m/s (high air damping), *m =* 2.61 × 10^−5^ kg, the equivalent acceleration noise of the mechanical thermal noise of the proof mass is 0.36 μg/Hz^0.5^. Obviously, the main noise is dominated by the resistance thermal noise of tunnel magnetoresistive sensor and circuit noise of the interface amplifier, and the prototype of tunneling magnetoresistive accelerometer has a large potential for improvement. Simultaneously, a drift stability curve of the z-axis tunneling magnetoresistive accelerometer based on Allan variance is shown in Figure 13 [24]. Allan variance analysis illustrates that the tunneling magnetoresistive accelerometer has a bias stability of 482 μg along z-axis sensitive direction. In summary, the above experimental results prove that the scheme of z-axis tunneling magnetoresistive accelerometer is feasible and effective and achieves a considerable performance.

## 6. Conclusions

The design, simulation, fabrication and experiments of a micromachined z-axis tunneling magnetoresistive accelerometer with electrostatic force feedback are proposed in the paper. The tunneling magnetoresistive accelerometer adopted the tunneling magnetoresistive effect with high sensitivity to measure magnetic field variation caused by input acceleration. A pair of lever-driven differential proof masses in the middle plane main structure is used for sensitiveness to acceleration and closed-loop feedback control. Compared with the traditional tunnel effect, the tunneling magnetoresistance effect with high sensitivity is directly generated by the multi-layer nano-film layer in the tunneling magnetoresistive sensors, which can be easily achieved with a multi-thin film deposition process. This avoids the micromachining requirement for a nano-gap between the tunnel tip and the plane. We constructed the finite element model of the mechanical structure and optimized the structural mode and mass ratio between inner and outer proof mass by the Ansys simulation. Simultaneously, we establish a magnetic field simulation model to analyze the magnetic field distribution and magnetic field change rate around the tunnel magnetoresistive sensor, as well as the effect of the location of tunneling magnetoresistive sensors, magnetic field intensity, and the dimension of permanent magnetic film on magnetic field sensitivity, which is beneficial to the achievement of maximum sensitivity. A standard deep dry silicon on glass (DDSOG) process is used to fabricate the micromachined z-axis tunneling magnetoresistive accelerometer which has a device dimension of 6400 μm (length) × 6400 μm (width) × 120 μm (height). The experimental results demonstrate the prototype has a maximal sensitivity of 8.85 mv/g along the z-axis sensitive direction under a gap of 1 mm. Simultaneously, Allan variance analysis illustrate that a noise floor of 86.2 μg/Hz^0.5^ is implemented in the z-axis tunneling magnetoresistive accelerometer, which proves that the scheme of z-axis tunneling magnetoresistive accelerometer is feasible and effective, and has great development potential in the future.

## Figures and Tables

**Table 1:** Structure parameters.

Parameter	Value	Parameter	Value
Inner proof mass (kg)	2.14 × 10^−5^	Thickness of main structure (μm)	120
Outer proof mass (kg)	4.68 × 10^−6^	U-suspension beam (length × width (μm))	465 × 15
Equivalent stiffness *k_e_* (N/m)	53.75	Torsional beam (length × width (μm))	519 × 15
Mode frequency *ω*_n_ (rad/s)	1435.61	Leverage (length × width (μm))	3600 × 150
Length 2*a* (μm)	3000	Gap d_1_ (between proof mass and feedback electrode (μm))	10
Width 2*b* (μm)	3000	Feedback electrode area (mm^2^)	15.36
Thickness 2*c* (μm)	500	Gap d_2_ (between tunnel magnetoresistive sensor and proof mass (μm))	1000
Inner proof mass (length × width (μm))	4000 × 4000	Moment density *M* (mT)	250
Outer proof mass (length × width (mm))	6400 × 6400	Maximum equivalent transform coefficien *k*_v_ (mV/mT)	3000

**Table 2:** The first six mode frequencies of the tunnel magnetoresistive accelerometer.

Modal	1	2	3	4	5	6
Frequency (Hz)	228.6	567.7	568.4	1051.7	1052.5	1516.2
